# Iodine status of pregnant women living in urban Johannesburg, South Africa

**DOI:** 10.1111/mcn.13236

**Published:** 2021-06-30

**Authors:** Sicelosethu S. Siro, Lizelle Zandberg, Jennifer Ngounda, Amy Wise, Elizabeth A. Symington, Linda Malan, Cornelius M. Smuts, Jeannine Baumgartner

**Affiliations:** ^1^ Centre of Excellence for Nutrition, Faculty of Health Sciences North‐West University (Potchefstroom Campus) Potchefstroom South Africa; ^2^ Department of Nutrition and Dietetics University of the Free State (UFS) Bloemfontein South Africa; ^3^ Department of Obstetrics and Gynaecology University of the Witwatersrand Johannesburg South Africa; ^4^ Empilweni Services and Research Unit University of the Witwatersrand Johannesburg South Africa; ^5^ Department of Life and Consumer Sciences University of South Africa Johannesburg South Africa; ^6^ Human Nutrition Laboratory Department of Health Sciences and Technology, Institute of Food, Nutrition and Health, ETH Zurich Zurich Switzerland

**Keywords:** iodine status, pregnant women, thyroid hormones, urban South Africa, urinary iodine concentration

## Abstract

Adequate intake of iodine is important during pregnancy because of its essential role in foetal growth and neurodevelopment. Data on iodine status of South African pregnant women are scarce, and the salt reduction policy implemented in 2016 may decrease iodine intake of South Africans. This cross‐sectional study assessed the iodine status of pregnant women residing in urban Johannesburg, South Africa. A total of 250 pregnant women were enrolled into the ‘Nutrition during Pregnancy and Early Development’ (NuPED) study and 312 pregnant women into the ‘Assessment of dried blood spot thyroglobulin in pregnant women to redefine the range of median urinary iodine concentration that indicates adequate iodine intake, South Africa’ (STRIPE‐SA) study and were included in this analysis. Urinary iodine concentration (UIC) was analysed in a spot urine sample. Thyroglobulin (Tg) was measured in serum, and thyroid‐stimulating hormone (TSH) and total thyroxine (tT4) were measured in dried blood spots. The median [interquartile range (IQR)] UIC of pregnant women was 144 (84–234) μg/L. Women in the first (*n* = 99), second (*n* = 262) and third (*n* = 174) trimester had a median UIC of 133 (81–316), 145 (84–236) and 156 (89–245) μg/L, respectively (*p* = 0.419). Median TSH, tT4 and Tg were 2.7 (2.3–3.2) mU/L, 202 (163–236) nmol/L and 9.2 (5.4–17.9) μg/L, respectively. Based on the median UIC, pregnant women residing in urban Johannesburg may be borderline iodine deficient. These findings highlight the need for ongoing monitoring of iodine status among vulnerable pregnant women, especially considering the recently introduced salt reduction policy in South Africa.

**Key messages**

- In South Africa, iodisation of table salt is mandatory, whereas iodisation of salt used for food production or animal consumption is voluntary.
- In 2016, the South African Government implemented a salt reduction policy, which may also affect the iodine intake of South Africans.
- Pregnant women are vulnerable to develop iodine deficiency due to increased requirements, but data on iodine status of South African pregnant women are scare.
- We showed that pregnant women living in urban Johannesburg may be borderline iodine deficient.
- There is a need for ongoing iodine status monitoring among pregnant women and other vulnerable groups in South Africa.

## 1 INTRODUCTION

Iodine is a vital element in the synthesis of thyroid hormones, which play a critical role in growth and brain development (Zimmermann, 2011). Iodine deficiency, though preventable, remains prevalent in all regions of the world (Zimmermann & Andersson, 2021). Iodine deficiency can lead to a range of adverse health outcomes depending on severity and occurrence in the human life cycle [Eastman & Zimmermann, 2018; World Health Organisation (WHO), 2013b]. The most serious consequences resulting from severe maternal iodine deficiency during pregnancy include stillbirth, foetal congenital anomalies and mental and growth restriction in offspring (Pearce et al., 2016). Recent observational studies have shown associations of mild‐to‐moderate iodine deficiency during pregnancy with impairments in neurodevelopmental outcomes in offspring (Hay et al., 2019). Pregnant women are susceptible to iodine deficiency because of increased demands to meet both increased maternal thyroid hormone production and foetal iodine needs and to compensate for increased renal iodine losses (Mioto et al., 2018; Pearce et al., 2016; Zimmermann, 2011). Therefore, the World Health Organisation (WHO) recommends that pregnant women consume 250 μg of iodine per day, which is 100 μg more than recommended for non‐pregnant and non‐lactating women (WHO et al., 2007).

Iodisation of salt is a fortification strategy that has been implemented worldwide to prevent iodine deficiency [Iodine Global Network (IGN), 2017]. Iodised salt is expected to have a protective effect in all population groups. However, some recent studies suggest that salt iodisation may not be adequate to meet increased requirements of pregnant women (Anaforoğlu et al., 2016; Mridha et al., 2017; Oral et al., 2016). In South Africa, a mandatory salt iodisation policy has been in effect since 1995 (Jooste et al., 1995), which led to the eradication of goitre (Jooste & Zimmermann, 2008). Even though the WHO recommends that monitoring of iodine status at a national level should be conducted every 3 to 5 years (WHO et al., 2007), the 2005 National Food Consumption Survey‐Fortification Baseline (NFCS‐FB‐I) was the last national survey that assessed iodine status in the South African population (Jooste et al., 2007). Results indicated that the iodine intake of South Africans is considered sufficient based on the assessment of median urinary iodine concentrations (UIC) in spot samples from women of childbearing age and school‐aged children. The iodine status of pregnant women was not assessed.

In 2016, South Africa implemented a salt reduction legislation that aimed at reducing sodium intake in the population by restricting the amount of salt added to processed foods. The use of iodised salt in food processing is not mandatory in South Africa; however, data from a recent study suggest that iodised salt is being used (Charlton et al., 2018). In a nationally representative sample of South African adults (*n* = 875) carried out prior to implementation of the salt reduction policy, participants who were already consuming <5 g/day of salt as recommended by WHO and targeted by legislation had a median UIC that was reflective of only borderline iodine sufficiency (102 μg/day) and did not meet the Estimated Average Requirements (EAR) for iodine intake (calculated from 24‐h urinary iodine excretion) (Charlton et al., 2018). Thus, to ensure optimal maternal and offspring health in South Africa, it is important to monitor the nutritional status of pregnant women. Therefore, this study aimed to assess the iodine status of pregnant women residing in urban Johannesburg, South Africa.

## 2 METHODS

### 2.1 Study design and participants

This cross‐sectional study was performed in pregnant women who participated in two studies, namely the ‘Nutrition during Pregnancy and Early Development’ (NuPED) study (*n* = 250) and the ‘Assessment of dried blood spot thyroglobulin in pregnant women to redefine the range of median urinary iodine concentration that indicates adequate iodine intake, South Africa’ (STRIPE‐SA) study (*n* = 312). The NUPED study is a prospective cohort study designed to follow‐up women during pregnancy and their infants postnatally. Pregnant women who participated in the NuPED study were recruited from primary healthcare clinics in Johannesburg between March 2016 and December 2017. The protocol of the study was previously published (Symington et al., 2018). For the purpose of the current study, data collected from the women at enrolment (<18 weeks gestation) were included. The STRIPE‐SA study has a cross‐sectional design and was conducted in pregnant women of any gestational age from September 2018 to February 2019 attending antenatal care at the Rahima Moosa Mother and Child Hospital (RMMCH) in Johannesburg of the Gauteng Province. Data collection for both studies was performed at the antenatal clinic of RMMCH antenatal clinic.

In both studies, pregnant women were included if they were between 18 and 39 years of age, born in South Africa or a neighbouring country, have lived in Johannesburg for at least 12 months, were able to communicate effectively in one of the local languages, non‐smoking, and expecting a singleton. In the NuPED study, pregnant women further had to be <18 weeks gestational age at recruitment.

In the NuPED study, women were excluded from participation if they reported use of illicit drugs, had a known non‐communicable disease (NCDs) such as diabetes, renal disease, history of high blood cholesterol and hypertension, and had a known infectious disease such as tuberculosis and hepatitis, or known serious illness such as cancer, lupus or psychosis. In the STRIPE‐SA study, pregnant women were excluded if they had a major medical illness, thyroid disease, HIV, and/or were taking major chronic medication (including antiretroviral drugs), have received iodine‐containing X‐ray/CT contrast agent or iodine‐containing medication within the last year, and if they were taking kelp and/or seaweed supplements. The NuPED study included HIV positive women to allow generalisation of results to the wider South African population that has a high prevalence of HIV (~36% of women aged 30–34 years) (Shisana et al., 2014).

### 2.2 Data collection

#### 2.2.1 Socio‐economic and demographic data

Socio‐economic and demographic data were collected from participants through a structured interview conducted by trained fieldworkers. Data collected included date and country of birth, marital status, educational level, employment status and beneficiaries of social grants. Living standards data were collected to allow classification according to the Living Standards Measure (LSM) developed by the South African Audience Reference Foundation (SAARF) (Haupt, 2016). This measure is widely used in South Africa to describe the socio‐economic status of the population (Labadarios et al., 2011). Women with an LSM score of 1–4, 5–7 or 8–10 were considered having a low, medium or high living standard, respectively. HIV status data were obtained from clinical records with consent from the participants. In the STRIPE‐SA study, we collected information on the use of iodine‐containing dietary supplements in the last 6 months and the use of iodised salt.

#### 2.2.2 Anthropometric measurements

Anthropometric measurements included height and weight. All measurements were performed twice and recorded to the nearest 0.05 kg for weight and 0.1 cm for height. Standardised methods of the International Society for the Advancement of Kinanthropometry (Marfell‐Jones et al., 2012) were used with a calibrated digital scale for weight (Seca Robusta 813) and a mobile stadiometer for height (Leicester Height Measure).

#### 2.2.3 Spot urine collection

Midstream spot urine samples (10–40 ml) were collected into clean plastic cups between 07:00 and 12:00 noon, and approximately, 5 ml was decanted into iodine‐free screw‐capped cups. The research team ensured that the urine samples were not used for any routine assessment using dipsticks to avoid potential contamination with iodine. Samples were aliquoted and stored on‐site at −20°C for a maximum of 7 days. Thereafter, samples were transported on dry ice to Centre of Excellence for Nutrition (CEN) laboratories in Potchefstroom, South Africa, for storage at −80°C until analysis.

#### 2.2.4 Blood sampling

Dried blood spots (DBS) were collected on Whatman 903 filter paper cards (Whatman Inc., USA). Whole blood was collected in the NuPED study by venous blood collection into an EDTA‐coated vacutainer (Becton Dickinson, Woodmead, South Africa) and in the STRIPE‐SA study by capillary blood collection via finger prick. Blood samples were spotted onto filter paper cards. Each filter paper card had six circles (spotting areas), and 50 μl of whole blood was spotted on each circle. The filter paper cards were allowed to dry at room temperature for 24 h, placed in zip lock bags with a desiccant and stored at −20°C for a maximum of 7 days before transportation on dry ice to CEN laboratories for storage at –80°C before shipment on dry ice to the Swiss Newborn Screening Laboratory, University Children's Hospital in Zurich, Switzerland for analysis. In the NuPED study, a serum sample was prepared from venous blood collected into a serum separator vacutainer tube (Becton Dickinson, Woodmead, South Africa) to obtain a serum sample, which was also stored on‐site at –20°C for a maximum of 7 days before transportation on dry ice to CEN laboratories for storage at –80°C before analysis.

### 2.3 Laboratory analysis

#### 2.3.1 UIC analysis

UIC in spot urine samples was measured in duplicate using the Pino modification of the Sandell–Kolthoff reaction with spectrophotometric detection at CEN (Jooste & Strydom, 2010; Pino et al., 1996). All analyses were done using nanopure grade water, and all laboratory glassware and plasticware were acid washed before use. Internal and external controls were used to ensure the quality of the analysis. Iodine concentrations in spot urine samples are expressed as median concentrations (μg/L). The median UIC cut‐off of <150 μg/L is used to define iodine deficiency in pregnant women (WHO, 2013b).

#### 2.3.2 TSH and tT4 analysis

Thyroid‐stimulating hormone (TSH) and total thyroxine (tT4) in DBS samples were measured at the Swiss Newborn Screening Laboratory, University Children's Hospital in Zurich, Switzerland. DBS‐TSH and DBS‐tT4 were analysed with the use of a time‐resolved dissociation‐enhanced lanthanide fluorescence immunoassay (DELFIA) on the genetic screening processor (GSP) and related kits (PerkinElmer, Turku, Finland), or with the use of a fluoro‐enzymatic immunoassay (FEIA) on the screening system NS2400 and related kits (Labsystem Diagnostics, Vantaa, Finland).

#### 2.3.3 Thyroglobulin

Thyroglobulin (Tg) was analysed in serum samples using the Q‐Plex™ Human Micronutrient Array (7‐plex, Quansys Bioscience, Logan, UT, USA) (Brindle et al., 2017) at the CEN. This fully quantitative chemiluminescent multiplex assay also includes Tg (Brindle et al., 2017). Tg was analysed for the NuPED participants and not for the STRIPE‐SA study. Analysis of Tg with this method was not within the scope of the STRIPE‐SA study.

### 2.4 Statistical analysis

Data processing and statistical analysis of data were performed using SPSS version 26 (IBM, Armonk, NY, USA). Raw data were captured in Microsoft Access, and 20% of all data were randomly checked for correctness. All UIC data were captured in Excel Windows XP (Microsoft, Seattle, WA, USA).

Baseline data from the NuPED study (<18 week's gestation) were pooled with the STRIPE‐SA data. Data were tested for outliers and normality using Q–Q plots, histograms and Shapiro–Wilk test. All data were non‐normally distributed and are expressed as medians [interquartile range (IQR)]. Categorical data are expressed as frequencies and percentages. Women were categorised by trimesters (first, second and third trimester), and the Kruskal–Wallis test was used to determine between‐group differences. Women were further grouped according to UIC status [(UIC < 150 μg/L and UIC ≥ 150 μg/L) and (UIC < 100 μg/L and UIC ≥ 100 μg/L)], and Mann–Witney *U* tests were performed to determine differences between groups. The Spearman's correlation was used to determine associations between continuous maternal characteristic and outcome variables. Analysis of covariance (ANCOVA) was performed to determine differences in TSH, tT4 and Tg between UIC categories [(UIC < 150 μg/L and UIC ≥ 150 μg/L) and (UIC < 100 μg/L and UIC ≥ 100 μg/L)], while controlling for maternal age and gestational age. TSH, tT4 and Tg were log‐transformed for univariate analysis.

### 2.5 Ethical considerations

This study was conducted according to the guidelines laid down in the Declaration of Helsinki, and all procedures involving research study participants were approved by the Human Research Ethics Committee of the North‐West University and the University of the Witwatersrand, Johannesburg. Permission to perform both the NuPED and STRIPE‐SA studies was given by the CEO of RMMCH, the RMMCH research review committee, the Gauteng Department of Health and the Johannesburg Health District's District Research Committee. Written informed consent was obtained from all participants before enrolment.

## 3 RESULTS

A total of 562 pregnant women were included in this analysis, of which 250 pregnant women were enrolled in the NuPED study and 312 where enrolled in the STRIPE‐SA study. Table 1 shows participant characteristics. Most women were of African descent (90.3%) and born in South Africa (67.6%). The median LSM score was 7 (6–8), and 60.4% of the women were classified as having a medium living standard. Participants in the NuPED study were significantly younger than the participants in the STRIPE‐SA study [27 (24–32) years and 31 (26–35) years, respectively; *p* < 0.001)]. The median gestational age was lower in participants of the NUPED than of the STRIPE study [4 (3–4) months and 7 (3–8) months, respectively, *P* < 0.001]. HIV prevalence in the NuPED study was 25.6%.

**Table 1:** Characteristics, urinary iodine concentration and thyroid function indicators of pregnant women participating in the NuPED and STRIPE studies

	Total sample (*n* = 562)	NuPED study sample (*n* = 250)	STRIPE study sample (*n* = 312)	*P* value^**^
	Median (IQR) or *n* (%)			
Characteristics				
Age (years)	30 (25–34)	27 (24–32)	31 (26–35)	<0.001
Gestational age (months)	4 (4–7)	4 (3–4)	7 (3–8)	<0.001
Weight (kg)	74.3 (63.3–87.0)	67.4 (58.0–77.8)	79.6 (68.7–92.9)	<0.001
Height (cm)	159 (155–163)	159.1 (155.1–162.4)	159.6 (155.0–163.9)	0.547
Country of birth, *n* (%)				
South Africa	371 (67.6)	172 (72)	199 (64.2)	<0.001*
Zimbabwe	129 (23.5)	60 (25.1)	69 (22.3)	
Lesotho	5 (0.9)	4 (1.7)	1 (0.3)	
Swaziland	4 (0.7)	3 (1.3)	1 (0.3)	
Other	40 (7.3)		40 (12.9)	
Years in South Africa, *n* (%)				
1–2 years	23 (4.2)	9 (3.8)	14 (4.5)	0.272*
>2–5 years	43 (7.8)	14 (5.9)	29 (9.4)	
≥5 years	482 (88)	216 (90.4)	266 (86.1)	
Ethnicity, *n* (%)				
Black	505 (90.3)	219 (88)	286 (92.3)	0.307*
Coloured	49 (8.7	28 (11.2)	21 (6.8)	
Indian	3 (0.5)	1 (0.4)	2 (0.6)	
White	2 (0.4)	1 (0.4)	1 (0.3)	
Education, *n* (%)				
None/primary	16 (3.2)	9 (3.6)	7 (2.7)	0.783*
Grade 8–10	71 (14.1)	37 (14.9)	34 (13.2)	
Grade 11–12	292 (57.8)	145 (58.2)	148 (57.6)	
Tertiary	126 (25)	58 (23.3)	68 (26.5)	
Employment, *n* (%)				
Unemployed	288 (51.5)	123 (49.4)	165 (53.2)	0.020*
Homemaker	6 (1.1)	—	6 (1.9)	
Self employed	28 (5.0)	10 (4.0)	18 (5.8)	
Wage earner	232 (41.5)	111 (44.6)	121 (39)	
Professional	1 (0.2)	1 (0.4)	—	
Other	4 (0.7)	4 (1.6)	—	
Marital status, *n* (%)^a^				
With partner	298 (53.4)	147 (59)	151 (48.9)	0.017*
Without partner	260 (46.6)	102 (41)	158 (51.1)	
Household grants, *n* (%)				
None	377 (68.9)	162 (66.9)	215 (70.5)	0.002*
Child support	135 (24.7)	69 (28.5)	66 (21.6	
Social relief	24 (4.4)	3 (1.2)	21 (6.9)	
Disability	2 (0.4)	2 (0.8)	—	
Old age pension	9 (1.6)	6 (2.5)	3 (1.0)	
LSM scores, *n* (%)				
1–4 (low)	40 (7.1)	17 (6.8)	23 (7.4)	0.782*
5–7 (medium)	338 (60.4)	148 (59.2)	190 (61.3)	
8–10 (high)	182 (32.5)	85 (34.0)	97 (31.3)	
HIV status				
HIV‐infected [*n* (%)]	64 (25.6)	64 (25.6)	0 (0)	
Urinary iodine and thyroid function indicators				
UIC (μg/L)	144 (84–234)	136 (75–235)	149 (91–233)	0.312
UIC < 150 μg/L, *n* (%)	285 (51)	133 (54)	152 (49)	0.302
TSH (mU/L)	2.7 (2.3–3.2)	2.3 (2.1–2.6)	3.0 (2.4–3.5)	<0.001
tT4 (nmol/L)	202 (168–236)	205 (168–238)	300 (160–236)	0.303
Tg (μg/L)	9.2 (5.4–17.9)	9.3 (5.4–17.9)	—	

Abbreviations: IQR, interquartile range; LSM, Living Standards Measure; Tg, thyroglobulin; tT4, total thyroxine; TSH, thyroid‐stimulating hormone; UIC, urinary iodine concentration.

^a^ Women who are with partner included those who reported living with a partner, being traditionally married and married, while without partner included those who reported being divorced, widowed or were single.

* Chi square‐test, *P* value significance set at *P* < 0.05.

^**^ Mann–Witney *U* test, *P* value significance set at *P* < 0.05.

**Figure 1:**
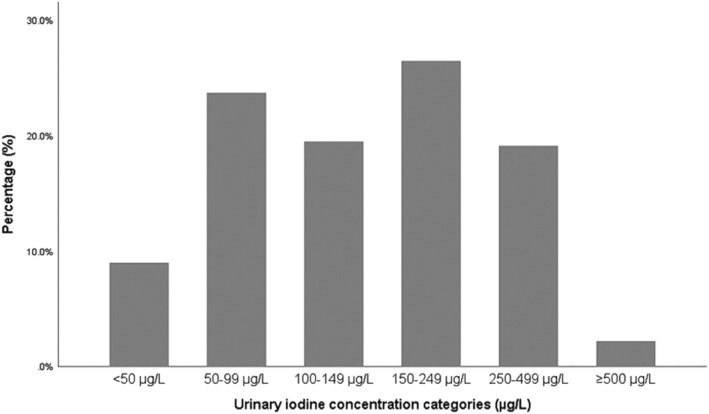
Frequency distribution of spot urinary iodine concentrations of pregnant women (*n* = 562) residing in Johannesburg, South Africa

The median (IQR) UIC of the pregnant women was 144 (84–234) μg/L (Table 1). Figure 1 shows the frequency distribution of spot UIC. About half of the women (52.2%) had a UIC < 150 μg/L (0–149 μg/L), and 32.7% had a UIC < 100 μg/L (0–99 μg/L). Only 2.2% had a UIC indicative of excessive iodine intake (UIC ≥ 500 μg/L). There was no significant difference in UIC between women assessed in the first trimester, second and third trimester (*p* = 0.419) (Table 2). The proportions of participants who had a UIC < 150 μg/L in the first, second and third trimester were 57.0%, 50.7% and 45.9%, respectively (Table 2).

**Table 2:** Differences in UIC, TSH and tT4 according to trimesters in pregnant women residing in Johannesburg, South Africa

	First trimester		Second trimester		Third trimester		*P* value*
	*N*	Median (IQR)	*N*	Median (IQR)	*N*	Median (IQR)	
UIC (μg/L)	99	133 (81–316)	262	145 (84–236)	174	155.7 (89–245)	0.419
TSH (mU/L)	66	2.4 (2.2–3.0)	170	2.5 (2.2–3.2)	146	3.0 (2.3–3.5)	0.010
tT4 (nmol/L)	69	178 (151–223)	177	211 (172–249)	172	194.8 (161–233)	0.001
Tg (μg/L)	72	9.0 (4.6–14.5)	177	9.5 (5.6–18.6)	—	—	0.159^**^

Abbreviations: IQR, interquartile range; Tg, thyroglobulin; tT4, total thyroxine; TSH, thyroid‐stimulating hormone; UIC, urinary iodine concentration.

* Kruskal–Wallis test, *P* value significance set at *P* < 0.05.

^**^ Mann–Witney *U* test, *P* value significance set at *P* < 0.05.

The use of iodised salt and the use of iodine‐containing supplements was assessed in the STRIPE‐SA study. A total of 97.1% (*n* = 303/310) of women reported to use iodised salt. Only 3.9% of women reported to take iodine‐containing supplements.

The median TSH was 2.7 (2.3–3.2) mU/L, and the median tT4 was 202 (163–236) nmol/L (Table 1). Both TSH and tT4 concentrations were significantly different between women assessed in the first, second and third trimester (*p* < 0.05) (Table 2). TSH was higher in the third trimester than in the first (*p* = 0.001) and second trimester (*p* = 0.01), whereas first and second trimester TSH concentrations were comparable (*p* = 0.55). Furthermore, tT4 was higher in the second trimester compared with the first trimester (*p* = 0.01). Maternal age positively correlated with TSH (*r* = 0.172, *p* = 0.001) and inversely correlated with tT4 (*r* = −0.127, *p* = 0.009). Gestational age positively correlated with TSH (*r* = 0.254, *p* < 0.001). There was no correlation between UIC, Tg, TSH and tT4. There was no significant difference in thyroid function indices between HIV positive and HIV negative participants. Furthermore, there was no difference in Tg, TSH and tT4 concentrations between women who had a UIC < 150 μg/L and women with a UIC ≥ 150 μg/L (*p* > 0.05), even after controlling for maternal age and gestational age. Also, when using a UIC cut‐off of <100 μg/L, no significant differences in Tg, TSH and tT4 were observed.

## 4 DISCUSSION

Adequate iodine nutrition during early embryonic and foetal development is important for growth and neurodevelopment, yet little is known about the iodine status of pregnant women living in South Africa. In this cross‐sectional study, the median UIC of pregnant women living in urban Johannesburg was 144 (84–234) μg/L, which is indicative of borderline iodine deficiency when considering the WHO stipulated UIC cut‐off of 150 μg/L (WHO, 2013b).

To our knowledge, there has been only one previous study that assessed the iodine status of pregnant women in a South African setting (conducted in 2012/2013). In contrast to our study, conveniently sampled pregnant women (*n* = 565) in the rural Limpopo province had a median UIC of 164 μg/L (92–291 μg/L), indicating sufficient iodine intake (Mabasa et al., 2018). The authors further showed that only 52.5% of the households used adequately iodised salt (salt iodine concentration ≥ 15 ppm), which is low in relation to the 90% goal of household penetration with iodised salt set by the WHO (UNICEF, 2015). Therefore, the authors attributed the sufficient iodine status in Limpopo to the presence of iodine in drinking water, which had a measured iodine concentration of 46.2 μg/L (Mabasa et al., 2018). However, only drinking water in the Limpopo and Northern Cape provinces of South Africa naturally contain iodine, whereas the contribution of iodine in drinking water to iodine status is neglectable in other provinces (Jooste et al., 2007). As such, if salt is not adequately iodised, iodine intake may be inadequate, especially in pregnant woman who have increased iodine requirements.

According to the most recent South African Demographic and Health Survey (SADHS) carried out in 2016, 89% of households in South Africa used adequately iodised salt (NDoH et al., 2019). In our study, we did not collect household salt samples for the analysis of salt iodine concentrations. However, 97% of the women who participated in the STRIPE study reported to buy iodised salt. Nonetheless, with the ongoing nutrition transition in South Africa, the consumption of discretionary salt (salt added either during cooking or at the table or both) is steadily declining, whereas the intake of non‐discretionary salt (salt in processed foods) is rising rapidly, especially in urban areas (Wentzel‐Viljoen et al., 2013). The South African salt fortification policy only mandates the iodisation of table salt (at a level of 35–65 ppm), whereas the iodisation of salt used for food production or animal consumption is voluntary (Jooste & Zimmermann, 2008). It is not known how many food producers in South Africa are currently using iodised salt. However, Charlton et al. (2018) recently reported a positive correlation between 24‐h urinary iodine excretion and 24‐h sodium excretion, which indicates that both discretionary and nondiscretionary salt are contributing to iodine intake in South Africans (Charlton et al., 2018). Furthermore, their study showed that there is a likelihood of inadequate iodine intake when the targeted salt intake of <5 g/day is met. Therefore, the salt reduction legislation may have lowered iodine intake in the South African population, which may explain the borderline iodine deficiency observed in our study conducted after implementation of the policy.

In countries undergoing salt reduction strategies, close monitoring of iodine status of the population, especially of vulnerable groups, is important to prevent the re‐emergence of iodine deficiency (WHO, 2013a). Thus, the lack of a recent national iodine survey in South Africa is concerning. The South African Nutrition and Health Survey (SANHANES) conducted in 2012 excluded iodine status assessment (Shisana et al., 2014) and the SADHS conducted in 2016 only assessed the adequacy of household salt iodisation (NDoH et al., 2019). Therefore, the most recent national survey that assessed iodine status of South Africans was conducted in 2005 (Jooste et al., 2007), and indicated adequate intake in women of reproductive age (UIC = 177 μg/L) and in school‐aged children (UIC = 215 μg/L). Based on these results from 15 years ago, South Africa is currently considered an iodine sufficient country.

In our study, we did not observe any differences in biomarkers of thyroid function between women who were classified as having inadequate and adequate iodine intake based on UIC. The reason probably being that UIC only reflects recent intake and cannot be used to classify individuals as iodine deficient or sufficient. As reference ranges for Tg, TSH and tT4 are poorly defined for pregnancy, we were unable to determine the prevalence of pregnant women with abnormal thyroid function in this study. The American Thyroid Association recommends the use of population‐based reference values when assessing TSH in pregnant women because of the differences that have previously been observed between different ethnic groups (Alexander et al., 2017). Furthermore, it has to be noted that the median UIC cut‐off of <150 μg/L to define iodine deficiency in pregnant women recommended by WHO is being debated. A recent pooled meta‐analysis by Nazeri and colleagues found that Tg concentrations was significantly higher in populations of pregnant women with a median UIC < 100 μL than in populations of pregnant women with a median UIC ≥ 100 μg/L. In contrast, Tg concentrations did not differ when comparing iodine sufficient and deficient populations of pregnant women based on the current threshold, indicating that physiological adaptations to maintain normal thyroid function only occur at a median UIC < 100 μg/L (Nazeri et al., 2020). Based on a median UIC < 100 μg/L, our sample of pregnant women would be considered iodine sufficient. However, previous studies have reported an association between mild‐to‐moderate iodine deficiency in pregnancy (classified as UIC 50–150 μg/L) and impaired mental development of offspring (Hynes et al., 2013; Markhus et al., 2018). Currently, the median UIC threshold at which offspring may be at risk for neurodevelopmental impairments is not known.

Limitations of our study include the lack of collection of household salt samples for the determination of salt iodine concentrations. Furthermore, the results from this study are not representative of the general population of pregnant women in South Africa but still represent a large urban area. Therefore, an iodine status survey in a nationally representative sample of pregnant women will be needed to assess whether iodine intakes of South African pregnant women are adequate and to determine whether there is a need to revise the salt iodisation policy. Such a revision could include an increase in salt fortification levels and/or mandating the iodisation of salt used for food production and in animal feeds, especially if future national surveys also indicate a decline in iodine intake in school‐aged children and women of reproductive age as a result of the salt reduction policy.

In conclusion, pregnant women residing in Johannesburg may be borderline iodine deficient. Thus, there is a need for ongoing iodine status monitoring in South African pregnant women, especially considering the recently introduced salt reduction policy.

## CONFLICTS OF INTEREST

The authors declare that they have no conflicts of interest.

## CONTRIBUTIONS

SSS, LZ and JB conceptualised and designed the study; EAS, AW, LZ, LM, MS and JB executed the study and collected data; SSS performed biochemical analyses and performed statistical analyses. SSS and JON wrote the first draft of the manuscript, and all authors read and edited the manuscript.

## Data Availability

The data that support the findings of this study are available from the corresponding author upon reasonable request.
